# A meta-analysis of randomized clinical trials on the impact of oral vitamin C supplementation on first-year outcomes in orthopedic patients

**DOI:** 10.1038/s41598-021-88864-7

**Published:** 2021-04-29

**Authors:** Kuo-Chuan Hung, Min-Hsien Chiang, Shao-Chun Wu, Ying-Jen Chang, Chun-Ning Ho, Li-Kai Wang, Jen-Yin Chen, Kee-Hsin Chen, Cheuk-Kwan Sun

**Affiliations:** 1 Department of Anesthesiology, Chi Mei Medical Center, Tainan City, Taiwan; 2 Department of Health and Nutrition, Chia Nan University of Pharmacy and Science, Tainan City, Taiwan; 3 Department of Anesthesiology, Kaohsiung Chang Gung Memorial Hospital, Chang Gung University College of Medicine, Kaohsiung City, Taiwan; 4 College of Health Sciences, Chang Jung Christian University, Tainan City, Taiwan; 5 Post-Baccalaureate Program in Nursing, College of Nursing, Taipei Medical University, Taipei, Taiwan; 6 Cochrane Taiwan, Taipei Medical University, Taipei, Taiwan; 7 Center for Nursing and Healthcare Research in Clinical Practice Application, Wan Fang Hospital, Taipei Medical University, Taipei, Taiwan; 8 Evidence-Based Knowledge Translation Center, Department of Nursing, Wan Fang Hospital, Taipei Medical University, Taipei, Taiwan; 9 Department of Emergency Medicine, E-Da Hospital, No.1, Yida Road, Jiaosu Village, Yanchao District, Kaohsiung City, 82445 Taiwan; 10 College of Medicine, I-Shou University, Kaohsiung City, Taiwan

**Keywords:** Health care, Medical research

## Abstract

This meta-analysis aimed at investigating the impact of oral vitamin C supplementation on the post-procedural recovery of orthopedic patients, including functional outcomes and complex regional pain syndrome type I (CRPS I). Literature search using the Medline, Cochrane Library, and Embase databases from inception till March 2021 identified seven eligible randomized controlled trials with 1,361 participants. Forest plot revealed no significant difference in the functional outcomes at 6–12 months [standardized mean difference (SMD) = −0.00, 95% CI − 0.19 to 0.18, 467 patients], risk of overall complications (RR = 0.98, 95% CI 0.68 to 1.39, 426 patients), and pain severity at 3–6 months (SMD = − 0.18, 95% CI − 0.49 to 0.12, 486 patients) between patients with and without oral vitamin C supplementation. Pooled analysis showed that vitamin C treatment reduced the risk of CRPS I regardless of dosage (RR = 0.46, 95% CI 0.25 to 0.85, 1143 patients). In conclusion, the current meta-analysis demonstrated that oral vitamin C supplementation may reduce the risk of complex regional pain syndrome type I but did not improve the functional outcomes in orthopedic patients. Nevertheless, because of the small number of trials included in the present study, further large-scale clinical studies are warranted to support our findings.

## Introduction

Reactive oxygen species (ROS), which are natural byproducts of normal metabolism, play an important role in homeostasis and cell signaling^1^. Although the production of ROS is regulated by antioxidant defense systems under normal physiological conditions^2^, excessive ROS generation and/or weakened antioxidant defense ability causes protein oxidation, lipid peroxidation, and nucleic acid oxidation^3^. Previous studies have revealed an essential role of ROS in inflammatory and neuropathic pain for which ROS scavengers showed potent antinociceptive effect^4^. Indeed, ROS have been implicated in many chronic pain conditions in clinical practice, including fibromyalgia^5^ and complex regional pain syndrome type I (CRPS I)^6^. Besides the association with skeletomuscular pain, ROS are known to adversely affect bone healing. An imbalance between ROS production and antioxidant defense systems^7^ results in oxidative stress that may be detrimental to healing after bone fracture^8^. In concert with this finding, a previous study reported acceleration of bone healing following long-bone fixative surgery through antioxidant vitamins A, E, and C as well as selenium administration^9^. In addition, previous studies have demonstrated an association between oxidative damage and age-related decline in skeletal muscle functional activity^10,11^.

Vitamin C (i.e., ascorbic acid), which is a water-soluble antioxidant and co-substrate of a large class of enzymes crucial for normal human growth and development^12^, is known to regulate gene expression through interacting with important transcription factors. Vitamin C is important for coping with all stressful conditions linked to inflammatory processes in which immunity is involved^13^. It is indispensable for collagen formation and bone development. In fact, vitamin C has a part to play in the formation of non-collagenous proteins and the development of cells (i.e., chondroblast, osteogenic, and mesenchymal) in the process of bone healing^14,15^. Vitamin C deficiency is known to delay healing after bone fractures^16^ and a higher dietary vitamin C intake is related to a reduction in femoral neck bone mineral density loss^17^. Consistently, several epidemiological^18,19^ and review^20^ studies have identified a positive association between bone mineral density and dietary vitamin C intake.

Despite the common belief that oxidative damage may be associated with chronic pain and impair the functional integrity of human skeletal muscle as well as bone healing^8^, the impact of vitamin C supplementation on functional outcomes in orthopedic patients remains unclear. Furthermore, there were ambiguous results from meta-analysis^21–25^ regarding the impact of vitamin C supplementation on CRPS I. The present meta-analysis aimed at assessing the impact of vitamin C supplementation on functional outcomes and updating the knowledge of the association of vitamin C supplementation with the risk of CRPS 1 in orthopedic patients.

## Materials and methods

### Guidelines and registration

This meta-analysis was conducted in compliance with the Preferred Reporting Items for Systematic Review and Meta-Analysis (PRISMA) guidelines^26^. The review protocol was registered with the PROSPERO international prospective register of systematic reviews (Registration No. CRD42020207721).

### Search strategy

Two authors (K.-C.H., C.-N.H.) independently searched the Medline, EMBASE, and Cochrane Controlled Trials Register (CENTRAL) databases for randomized controlled trials (RCTs) which investigated the clinical outcomes in orthopedic patients with and without vitamin C supplementation. The last search was performed on March 27, 2021. To identify relevant articles, search keywords used controlled vocabulary (MeSH or Emtree) and text words including: "bone fracture*" or "fracture*" or "orthopedic*" or "orthopaedic*" or "bone injury" or "fracture healing" or "bone healing" or "bone*" or "Fractures, Bone [MeSH]" or "Orthopedic Procedures [MeSH]" or "Orthopedics [MeSH]" or "vitamin C" or "L-Ascorbic" or "Ascorb*" or "vit c" or "Magnorbin" or "hybrin" or "ascorbic acid [MeSH]". Results were combined using the Boolean operator “AND” with the search terms. References from relevant studies were searched to find additional studies. No publication date was applied, but only trials published in English were included.

### Selection criteria

Two reviewers (K.-H.C., L.-K.W.) independently examined the abstracts of the acquired articles to identify potentially eligible studies. The PICO criteria for eligibility of RCTs for the current study included: 1) Population: adult patients (i.e., ≥ 18 years) who had orthopedic disorders that were treated conservatively or surgically; 2) Intervention: vitamin C was given as an intervention rather than a control through oral or intravenous route; 3) Comparison: placebo or no therapy; and 4) Outcome: any outcomes such as risk of CRPS I and/or functional outcomes (i.e., ≥ 3 months). There were no restrictions on the timing of administration and dosage. The exclusion criteria were (1) non-RCT studies including before-and-after studies, (2) studies that focused on pediatric population because of the difficulty in outcome assessment, (3) those in which information regarding dosage of vitamin C or outcomes was unavailable, and (4) those adopted vitamin C as a placebo. Two authors (J.-Y.C., Y.-J.C.) independently investigated the selected trials for the final analysis. In the situation of disagreements, a third author (C.-K.S.) was involved until a consensus was reached.

### Data extraction

Two authors (S.-C.W., M.-H.C.) extracted relevant data from each selected trial and entered them into predefined databases. Divergences were resolved by discussion. If the included studies did not report data on primary or secondary outcomes, the corresponding authors were contacted for further information. The following data were extracted from each trial: author, publication year, study setting, patient characteristics, sample size, orthopedic procedures, dosage of vitamin C, incidence of CRPS I, functional outcomes, intensity of pain, and adverse events.

### Primary outcome, secondary outcomes, and definitions

Clinical outcomes of the present meta-analysis were defined as those after a follow-up of 3–12 months. The primary endpoint was the impact of vitamin C supplementation on the functional outcomes following bone injury or orthopedic procedures, while the secondary outcomes included the risk of CRPS I, pain score, and risk of overall complications. The definition of functional outcomes and criteria for CRPS I were defined according to the criteria of each study.

### Assessment of risk of bias for included studies

Two authors (M.-H.C., Y.-J.C.) assessed the risk of bias for each trial using the criteria outlined in the Cochrane Handbook for Systematic Reviews of Interventions^27^. Disagreements were solved by discussion. The overall risk of bias of all included studies and the risk of bias of individual studies were analyzed. We rated the potential risk of bias by applying a rating of “low”, “high,” or “unclear” to each trial.

### Statistical analysis

Instead of using the fixed model, a random effects model was adopted to calculate the risk ratios (RRs) with 95% confidence intervals (CIs) for dichotomous outcomes taking into account probable study heterogeneity arising from differences in investigators and settings. Pooling of dichotomous data and computation of pooled RRs with 95% CIs was achieved by the Mantel–Haenszel (MH) method. The standardized mean difference (SMD) was used to express the selected effect size for continuous outcome. Assessment of heterogeneity (low: < 50%; moderate to high: 50% to 75%; high: > 75%) was attained by the application of I^2^ statistics. To identify the possible influence of a single study on the overall results, sensitivity analyses were conducted to evaluate the impact of a single clinical trial on the overall findings by deleting one study at a time from the meta-analysis. In addition, subgroup analyses were conducted based on the indications for the procedures (i.e., fracture subgroup vs. non-fracture subgroup). We used funnel plots to assess the potentials of publication and reporting bias if ten or more studies were available for the analysis of a particular outcome. The result was considered significant for a probability value (*p*) less than 0.05 for all analyses. For data synthesis, we utilized the Cochrane Review Manager (RevMan 5.4; Copenhagen: The Nordic Cochrane Center, The Cochrane Collaboration, 2014).

## Results

### Study selection

**Figure 1:**
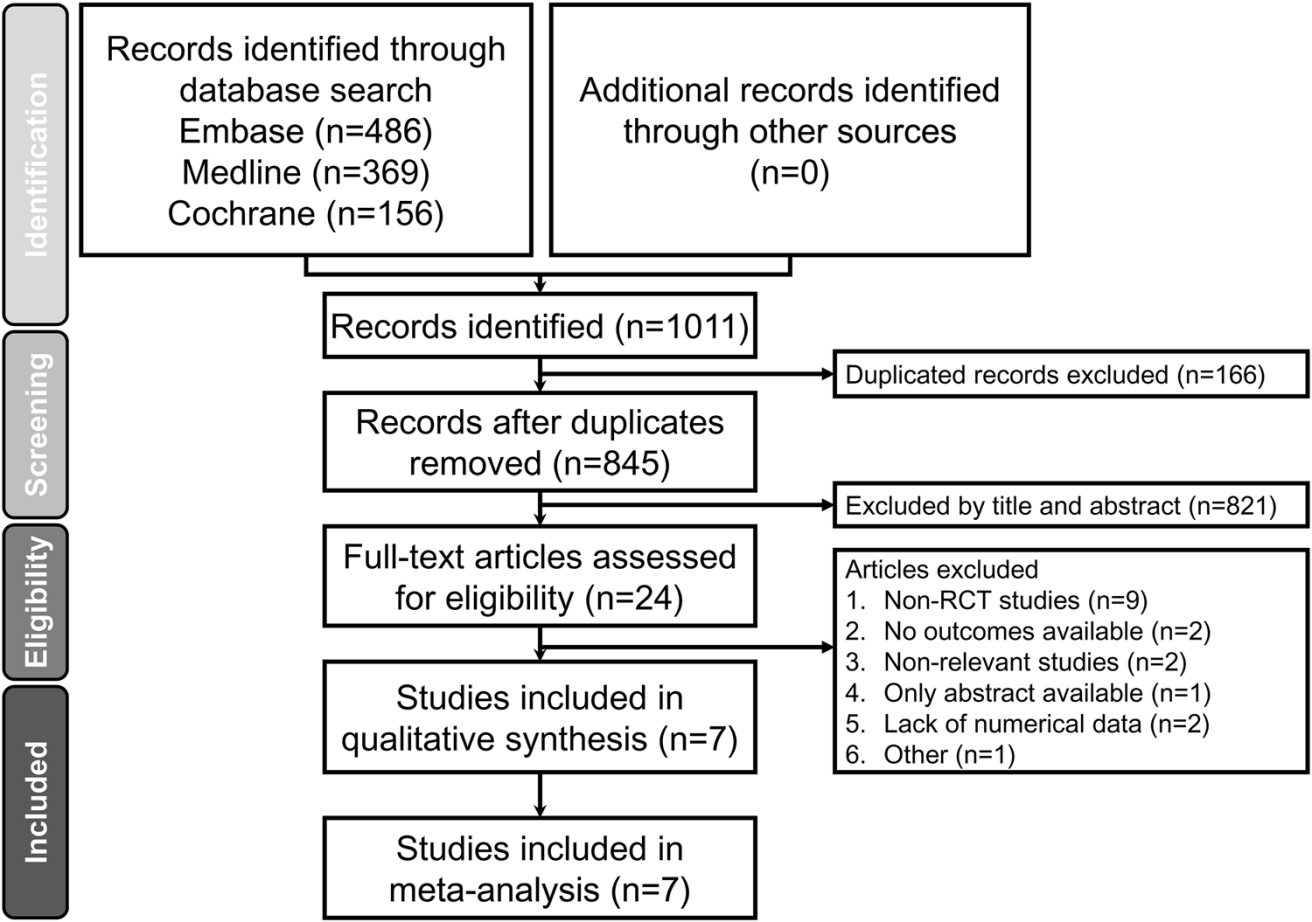
PRISMA flowchart for selecting eligible studies. RCT: randomized controlled trial.

A flowchart summarizing the reasons for study exclusion according to the Preferred Reporting Items for Systematic reviews and Meta-Analyses (PRISMA) guidelines as well as an illustration of the full electronic search strategy using the Medline database are shown in Fig. 1 and Supplemental Fig. 1, respectively. Of a total of 1,011 potentially eligible trials identified from the databases, 166 were removed due to duplication and 821 records were excluded after reviewing their titles and abstracts. Of the remaining 24 studies deemed eligible with the full text surveyed, 17 studies were deleted because of non-RCT studies (n = 9), no available outcomes (n = 2), irrelevance (n = 2), availability of merely an abstract (n = 1), shortage of numerical data (n = 2), and non-English publication (n = 1). Overall, seven RCTs in total were included in the present meta-analysis (Fig. 1).

### Characteristics of included studies

Seven RCTs including 1,361 participants published between 1999 and 2021 were analyzed^28–34^. The study characteristics are described in Table 1. Vitamin C was given orally in all trials^28–33^ with a duration ranging from 40 to 55 days. The dosage of vitamin C was 1000 mg daily in three studies^28,30,34^ and 500 mg daily in three trials^29,31,33^, while one study compared the effects of three different daily doses (i.e., 200, 500, 1000 mg)^32^. The follow-up was 12 months in six studies^28,29,31–34^ and 12 weeks in the other study^30^. Three studies focused on patients with wrist fracture^29,32,33^, while the other four investigated patients with total knee replacement (n = 2)^28,34^, foot and ankle trauma (n = 1)^30^, and lumbar spine surgery (n = 1)^31^, respectively. For a study that divided patients with a wrist fracture into displaced and non-displaced sub-group^29^, we separated the two groups for convenience of statistical analysis. According to the indications for the procedures, subgroup analysis was performed on the seven studies included in the current meta-analysis that were divided into the fracture (i.e., three studies on wrist fracture^29,32,33^ and one study on ankle fracture^30^) and non-fracture (i.e., one study on lumbar spine stenosis^31^ and two studies on total knee arthroplasty^28,34^) subgroups.

**Table. 1:** Characteristics of included studies (n = 7).

	Orthopedic procedures or location of fractures	Patient number (Vit C vs. Placebo)	Female (%) (Vit C vs. Placebo)	Vitamin C dosage (daily)	Time of administration (days)	Scores for Functional outcomes	Criteria for CRPS I diagnosis	Follow-up
Behrend 2019^28^	Total knee replacement	48 vs. 47	52% vs. 48%	1000 mg	55	FJS-12	NA	12 months
Ekrol 2014^29^	Wrist fractures	124 vs. 125	74% vs. 72.5%	500 mg	55	DASH score	Atkins	52 weeks
Jain 2019^30^	Foot and ankle trauma	30 vs. 30	16.7% vs 33.3%	1000 mg	42	FAI scale	NA	12 weeks
Lee 2017^31^	Lumbar spine surgery	62 vs. 61	33.9% vs. 37.7%	500 mg	45	ODI	NA	12 months
Zollinger 2007^32^	Wrist fractures	328 vs. 99	83% vs. 80%	200, 500, 1500 mg	55	NA	Veldman	12 months
Zollinger 1999^33^	Wrist fractures	52 vs. 63	78% vs. 80%	500 mg	55	NA	Veldman	12 months
Jacques 2021^34^	Total knee replacement	153 vs. 139	60% vs. 70%	1000 mg	40	NA	Budapest	12 months

Vit. C: vitamin C; CRPS: complex regional pain syndrome; NA: not available; FJS-12: Forgotten Joint Score-12; DASH score: The Disabilities of the Arm, Shoulder and Hand Score; FAI scale: Foot and ankle outcome instrument scale; ODI: Oswestry Disability index.

### Risk of bias assessment

**Figure 2:**
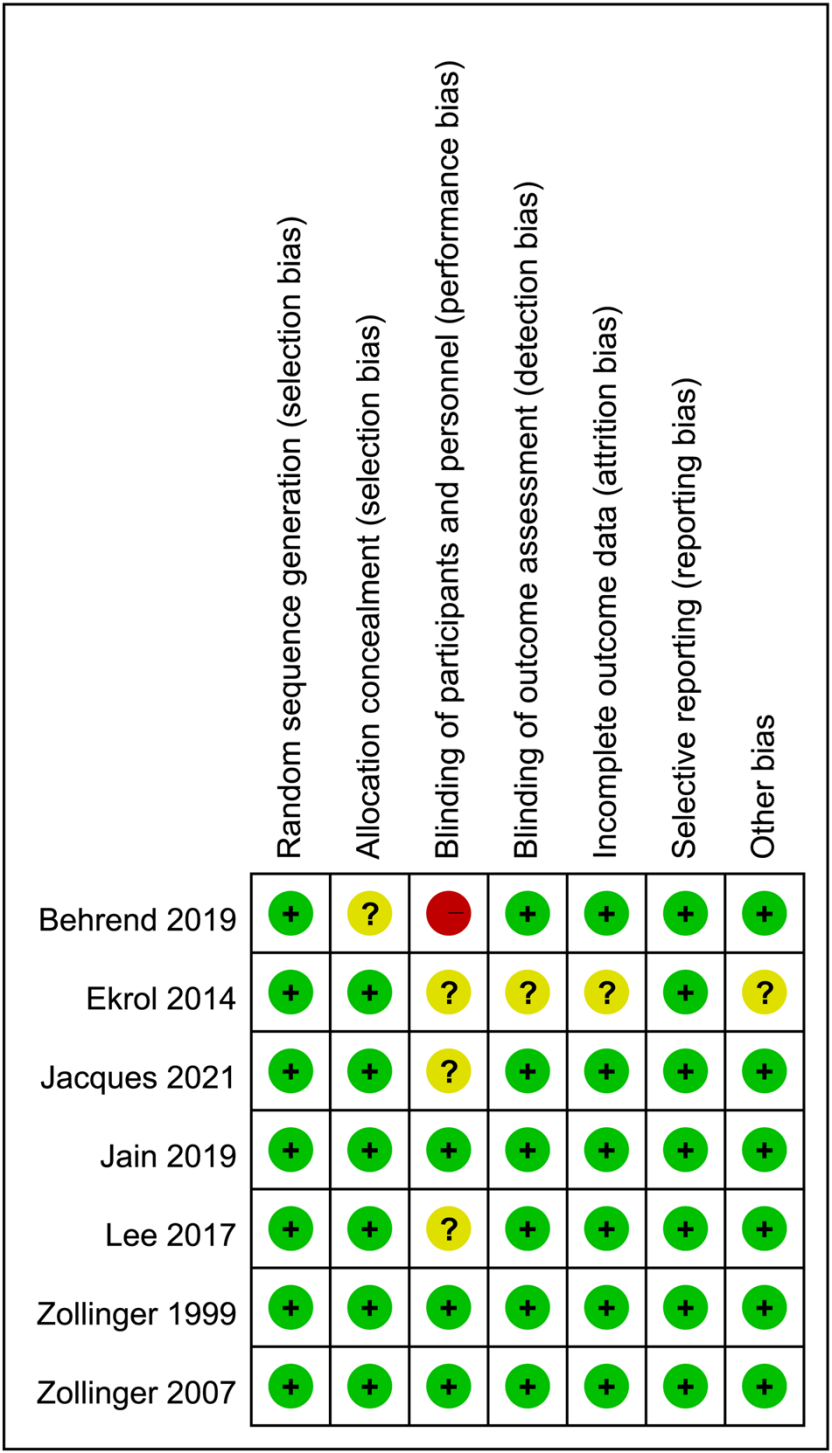
Risks of bias of individual studies.

The risks of bias of individual studies are summarized in Fig. 2. All included studies were found to give sufficient details about randomization and were assigned a low risk of allocation bias^28–34^. The risk of performance bias of three trials was considered low because both participants and investigators were blinded to the treatment (e.g., all capsules had the same appearance and taste)^30,32,33^. Other risks of bias including attrition bias, measurement bias, reporting bias, and overall bias were also regarded as low in most studies.

### Improvement in functional outcomes at 3–6 months

**Figure 3:**
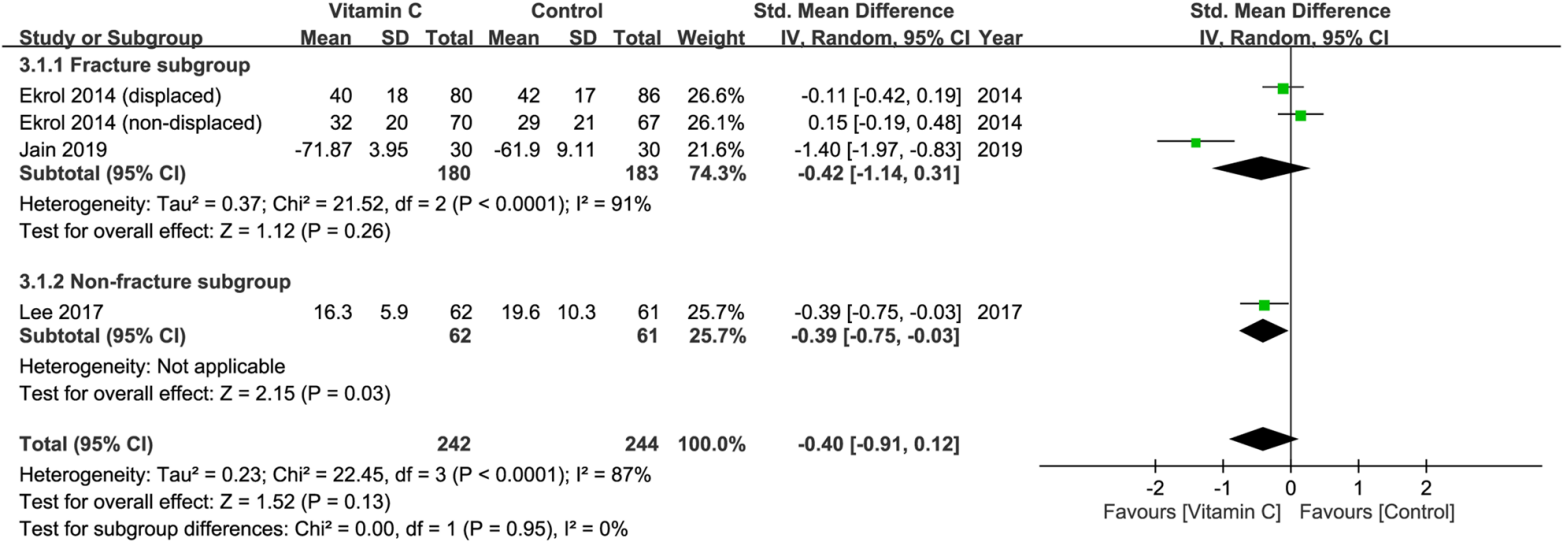
Forest plot for the comparison of function outcomes at 3–6 months between vitamin C and placebo groups. CI, confidence interval; IV, inverse variance; Std., standardized.

Three studies recruiting a total of 486 patients (vitamin group, n = 242 vs. placebo group, n = 244) were eligible for the analysis^29–31^. A forest plot demonstrated no significant difference in functional outcomes at 3–6 months between both groups (SMD = − 0.40, 95% CI − 0.91 to 0.12, *p* = 0.13; I^2^ = 87%) (Fig. 3). Subgroup analysis did not demonstrate a significant difference between the fracture and non-fracture subgroups (*p* = 0.95). Omitting certain trials also did not significantly impact the outcomes on sensitivity analysis.

### Improvement in functional outcomes at 6–12 months

**Figure 4:**
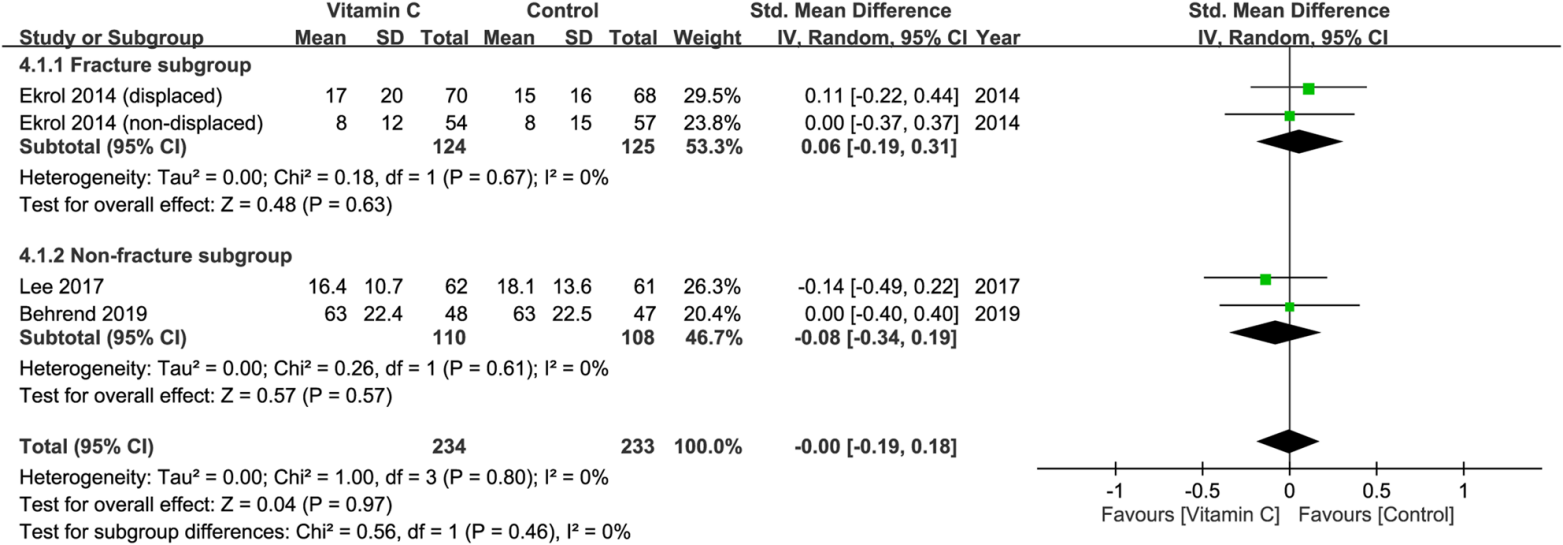
Forest plot for the comparison of function outcomes at 6–12 months between vitamin C and placebo groups. CI, confidence interval; IV, inverse variance; Std., standardized.

Three studies with a total of 467 patients (vitamin group, n = 234 vs. placebo group, n = 233) were available for the analysis^28,29,31^. Inspection of the forest plot revealed no significant difference in functional outcomes at 6–12 months between both groups (SMD = − 0.00, 95%: CI − 0.19 to 0.18, *p* = 0.97; I^2^= 0%) (Fig. 4). No significant difference between fracture and non-fracture subgroups was noted on subgroup analysis (*p* = 0.46). In addition, sensitivity analysis did not show significant impact on outcome by omitting certain trials.

### Risk of CRPS I at follow-up of 12 months in patients receiving oral vitamin C regardless of dosage

**Figure 5:**
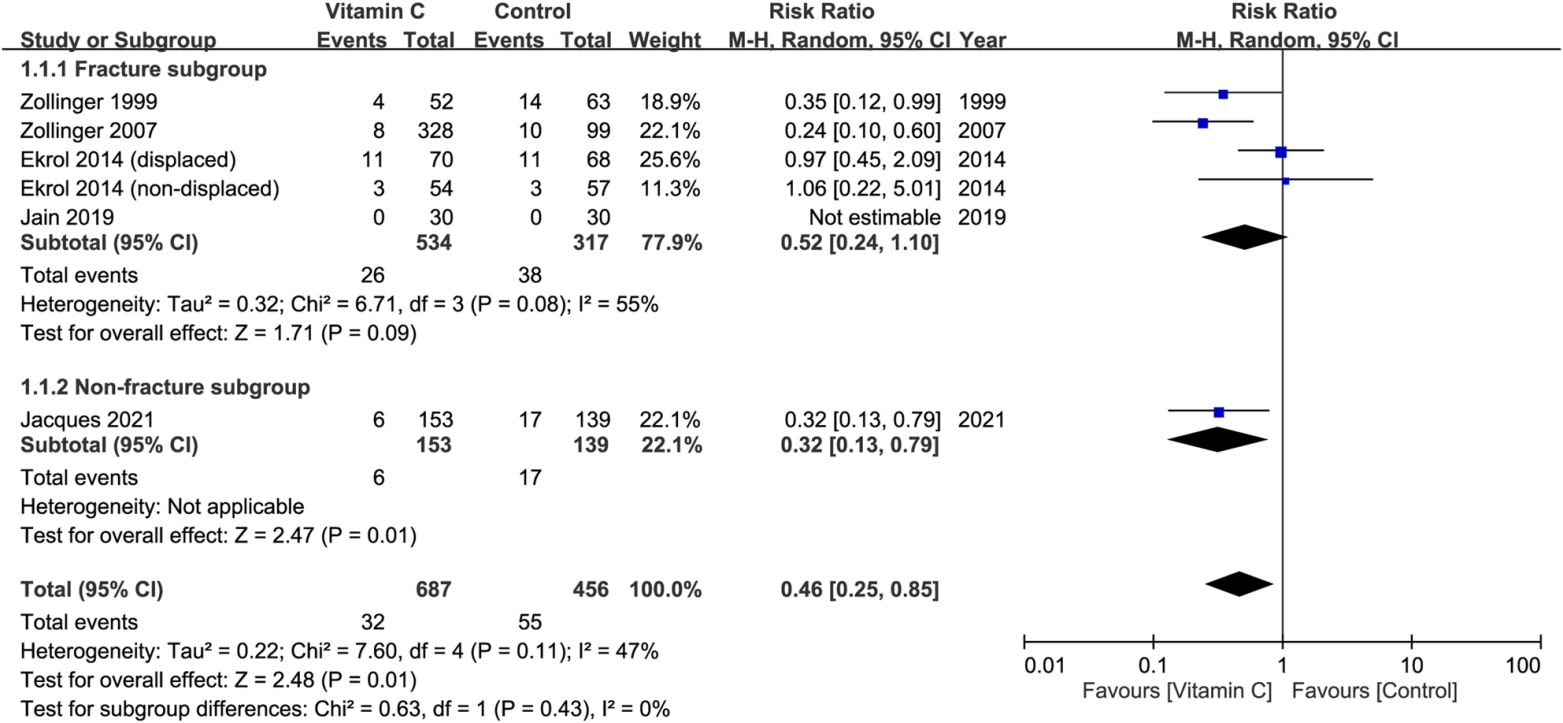
Forest plot for the comparison of risk of complex regional pain syndrome I between vitamin C and placebo groups at follow-up of 12 months. CI, confidence interval; M–H, Mantel–Haenszel.

Five studies involving a total of 1,143 patients (vitamin group, n = 687 vs. placebo group, n = 456) contained information for CRPS I risk assessment^29,30,32–34^. The definition of ‘CRPS I’ varied across the included studies (Table 1). The dosage of vitamin C varied from 200 to 1500 mg (Table 1). Pooled analysis showed that the risk of CRPS I was lower in patients with vitamin C treatment compared to those without (RR = 0.46, 95% CI 0.25 to 0.85, *p* = 0.01; I^2^ = 47%) (Fig. 5). Subgroup analysis showed no significant difference between the fracture and non-fracture subgroups (*p* = 0.43). However, inconsistency of outcome was noted on sensitivity analysis when three trials were removed one at a time^32–34^.

### Risk of CRPS I at follow-up of 12 months in patients receiving oral vitamin C ≥ 500 mg daily

**Figure 6:**
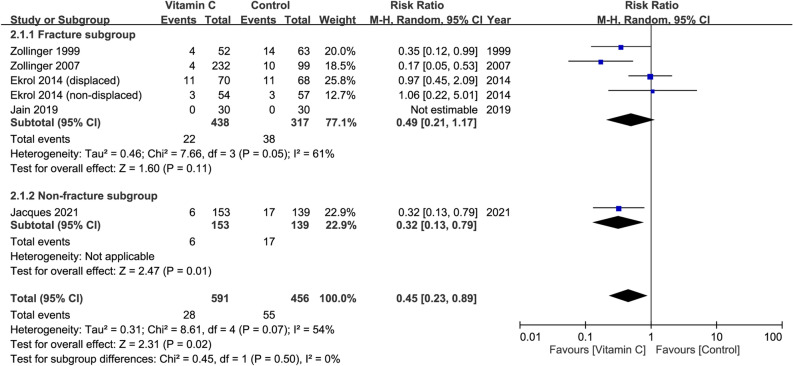
Forest plot for the comparison of risk of complex regional pain syndrome I between vitamin C (dosage ≥ 500 mg daily) and placebo groups at follow-up of 12 months. CI, confidence interval; M–H, Mantel–Haenszel.

Five studies with a total of 1,047 patients (vitamin group, n = 591 vs. placebo group, n = 456) were available for the analysis of risk of CRPS I in patients receiving vitamin C ≥ 500 mg daily^29,30,32–34^. The dosage of vitamin C varied from 500 to 1500 mg (Table 1). Consistent the overall outcome regardless of dosage, pooled analysis also demonstrated a reduced risk of CRPS I in patients with relatively high-dose vitamin C supplementation compared to those without (RR = 0.45, 95% CI 0.23 to 0.89, *p* = 0.02; I^2^ = 54%) (Fig. 6). Subgroup analysis of the fracture and non-fracture subgroups demonstrated no significant difference (*p* = 0.5). On the other hand, sensitivity analysis showed an inconsistent outcome when three trials^32–34^ were removed one at a time.

### Severity of pain 3–6 months after surgery or trauma

Three studies enrolling a total of 486 patients (vitamin group, n = 242 vs. placebo group, n = 244) provided data on pain severity for the analysis^29–31^. A forest plot demonstrated no significant difference in mean pain score at 3–6 months in the vitamin C group compared with that in the placebo group (SMD = − 0.18, 95% CI − 0.49 to 0.12, *p* = 0.25; I^2^ = 64%) (Supplemental Fig. 2). Subgroup analysis between the fracture and non-fracture subgroups revealed no significant difference (*p* = 0.93). Besides, sensitivity analysis gave no evidence of a significant impact on outcome by omitting certain trials.

### Overall complications at 3–6 months after surgery or trauma

Two studies involving a total of 426 patients (vitamin group, n = 212 vs. placebo group, n = 214) reported the incidences of complications for the analysis^29,31^. The pooled RR of complications at 3–6 months was 0.98 (95% CI 0.68 to 1.39, *p* = 0.89, I^2^ = 0%) (Supplemental Fig. 3). The findings showed no significant association between the use of oral vitamin C supplementation and the risk of complications following surgery or trauma. Comparison between the fracture and non-fracture subgroups also demonstrated no significant difference on subgroup analysis (*p* = 0.77).

## Discussion

Despite the potential therapeutic effects of oral vitamin C supplementation on osteoporosis, bone mineral density, and physical performance^35,36^, our findings demonstrated no significant differences in the functional outcomes, pain score, and overall complications between patients with oral vitamin C supplementation and those without following orthopedic procedures. In addition, although previous meta-analyses reported that vitamin C supplementation may decrease the risk of CRPS I^21,22,24,25^, results from the other authors did not share the same conclusion^23^. With the incorporation of the latest data, our study showed a lower risk of CRPS I in patients with oral vitamin C treatment compared to those without.

In a meta-analysis of observational studies, an increased dietary vitamin C intake was associated with a lower risk of hip fracture and osteoporosis as well as a higher bone mineral density at femoral neck and lumbar spine^35^. In addition, recent studies suggested a positive correlation between vitamin C intake and physical performance in the elderly^37^. Consistently, another study examining the relationship between antioxidant vitamin intake (e.g., vitamin C, vitamin E, retinol, and β-carotene) and physical performance in the elderly demonstrated significant positive correlations between most antioxidants, particularly vitamin C, and a higher skeletal muscular strength (e.g., knee extension) and physical performance^36^. Because muscle strength recovery is a key factor affecting the functional outcome of post-fracture patients^38–40^, vitamin C may have a role to play in reinforcing functional recovery after fracture. Nevertheless, the current study did not show evidence supportive of this hypothesis.

CRPS I, which is an uncommon complication of orthopedic surgery with a female predominance, has an incidence of 10.1–22.2%^32,33^. CPRS I is characterized by unexplained pain, edema, swelling, sudomotor and motor dysfunctions (e.g., vasomotor instability) and loss of joint mobility. Early treatment is critical for a good prognosis^41^. Previous clinical and animal studies have demonstrated a reduced incidence of CRPS I after trauma and surgery through the oral vitamin C administration^29,42,43^. In concert with this finding, although a number of meta-analyses reported a decreased risk of CRPS I following orthopedic procedures after the use of vitamin C^21,22,24,25^, other meta-analyses did not share the same findings^23^. Therefore, the association between vitamin C supplementation and the risk of CRPS remains unclear.

The limitations of previous meta-analyses investigating the correlation between oral vitamin C supplementation and the risk of CRPS I are summarized in supplemental Table 1. For instance, one meta-analysis^22^ included only one RCT^44^ and two non-RCT studies^42,45^ without incorporating the results of some RCTs^29,33^. Although another meta-analysis focused on RCTs^21^, the included trials involved patients subjected to different forms of treatment (i.e., conservative^33^ vs. conservative or surgery^29,32^), inclusion and exclusion criteria, and criteria for diagnosing CRPS I. Despite the finding of an association between oral vitamin C supplementation and a decreased risk of CRPS I^21^, the application of fixed effects model based on the assumption of homogeneity of the included trials sampled from the same population^46^ instead of random effects model in that study may have biased their results. In contrast, using the random effects model and evidence from the latest studies, our investigation demonstrated a significant positive association between oral vitamin C supplementation and a reduced risk of CRPS I. However, the inconsistent outcome of sensitivity analysis implicated a lack of robustness of the results that warrant further large-scale clinical studies for elucidation.

Patients who are severely injured or after major surgeries are subject to a high level of systemic stress that causes an excessive production of ROS and a significant consumption of endogenous antioxidants^7,47^. Such an imbalance between ROS generation and antioxidant reinforcement perpetuates the oxidative stress, resulting in reduction/oxidation (redox) dysregulation, cellular perturbation, organ dysfunction, and systemic disorders^7^. A retrospective study on 4,294 trauma patients demonstrated that the implementation of a high-dose antioxidant protocol including intravenous vitamin C (1000 mg every eight hours) was associated with a reduced incidence of respiratory failure, surgical site infections, abdominal wall complications, and overall infectious complications^47^. Consistently, a previous meta-analysis on a total of 19 RCTs involving 2,008 patients showed a significant decrease in the incidence of atrial fibrillation, ventilation time, length of ICU and hospital stay in those receiving intravenous or oral vitamin C supplementation^48^. Nevertheless, the pooled RR of complications at 3–6 months in the current study was 0.98 (95% CI 0.68 to 1.39, *p* = 0.89, I^2^ = 0%) (supplemental Fig. 3), indicating that the use of vitamin C was not associated with a lower risk of complications. The difference in findings between the present investigation and previous studies may be partly explained by the variations in patients’ condition, the magnitude of surgeries, the surgical techniques, and the incidence of inflammation and ischemia/reperfusion injury that the patients experienced^7^. Therefore, the inclusion of trials on relatively minor procedures in patients without major trauma or hemodynamic instability in the current meta-analysis may contribute to the lack of significant benefit of oral vitamin C supplementation in terms of reduction in postoperative complications. Additionally, in contrast to the current investigation that pinpointed the therapeutic benefit of oral vitamin C supplements, previous studies investigated patients receiving either pure intravenous^47^ or mixed intravenous and oral^48^ vitamin C. As intravenous vitamin C administration can achieve a circulating concentration up to 70-fold higher than that through the oral route at the maximum tolerable dose^49^, the lack of effectiveness of oral vitamin C is possible in this clinical setting.

The current meta-analysis had its limitations that need to be taken into account for accurate interpretation of its findings. First, our study included a relatively small number of RCTs on patients receiving different orthopedic procedures. Second, we only investigated the effects of oral vitamin C supplementation on the prevention rather than the treatment of CRPS I because there was no study on the latter. Third, review of the included trials of the present study^29^ revealed that the incidence of CRPS I appeared to vary with the time after surgery; the incidence at postoperative 6 weeks was higher than that at one year. Therefore, the choice of postoperative 12 months as the time of outcome assessment in the current study may result in underestimation of the beneficial effect of vitamin C. Nevertheless, only one of the included studies provided the incidence of CRPS I at three months after procedures so that the short-term effectiveness of vitamin C supplementation could not be assessed. Fourth, although CRPS I mostly affects women^50^, all five of our included trials recruited males who may have biased the findings. Finally, there are different criteria for the diagnosis of CRPS I (e.g., Budapest, Atkins and Veldman criteria)^51^. Of the five studies addressing CRPS I in the present study, two adopted the Veldman criteria, one used the Atkins criteria, and one applied the Budapest criteria. Hence, the small number of studies and the different criteria used may potentially bias our results.

In conclusion, our systematic review of clinical trials investigating the effects of oral vitamin C supplementation on recovery in patients after orthopedic procedures demonstrated no significant impact of vitamin C on the functional outcomes at 3–6 months or 6–12 months, the risk of overall complications and the severity of pain at 3–6 months after orthopedic procedures. However, patients with oral vitamin C supplementation regardless of dosage had a lower risk of CRPS I on follow-up at 12 months compared to that in those without. Nevertheless, the small number of randomized clinical trials included in this study warrants further large-scale studies to support our findings.

## Supplementary Information

Supplementary Information
