# Supplementary material for: A meta-analysis of randomized clinical trials on the impact of oral vitamin C supplementation on first-year outcomes in orthopedic patients
Source: Sci Rep. 2021 Apr 29;11:9225. doi: 10.1038/s41598-021-88864-7 (PMC8085077; doi:10.1038/s41598-021-88864-7)
Supplement: Supplementary file 1 — Supplementary Information [file 41598_2021_88864_MOESM1_ESM.docx]

Review

A meta-analysis of randomized clinical trials on the impact of oral vitamin C supplementation on first-year outcomes in orthopedic patients

Kuo-Chuan Hung^1,2^, Min-Hsien Chiang^3^, Shao-Chun Wu^3^, Ying-Jen Chang^1,4^, Chun-Ning Ho^1^, Li-Kai Wang^1,2^, Jen-Yin Chen^1^, Kee-Hsin Chen^5,6,7,8^* and Cheuk-Kwan Sun^9,10,^*

^1^ Department of Anesthesiology, Chi Mei Medical Center, Tainan city, Taiwan;

^2^ Department of Health and Nutrition, Chia Nan University of Pharmacy and Science, Tainan city, Taiwan

^3^ Department of Anesthesiology, Kaohsiung Chang Gung Memorial Hospital, Chang Gung University College of Medicine, Kaohsiung city, Taiwan

^4^ College of Health Sciences, Chang Jung Christian University, Tainan city, Taiwan

^5^ Post-Baccalaureate Program in Nursing, College of Nursing, Taipei Medical University, Taipei, Taiwan

^6^ Cochrane Taiwan, Taipei Medical University, Taipei, Taiwan

^7^ Center for Nursing and Healthcare Research in Clinical Practice Application, Wan Fang Hospital, Taipei Medical University, Taipei, Taiwan

^8^ Evidence-based Knowledge Translation Center, Department of Nursing, Wan Fang Hospital, Taipei Medical University, Taipei, Taiwan

^9^ Department of Emergency Medicine, E-Da Hospital, Kaohsiung city, Taiwan

^10^ College of Medicine, I-Shou University, Kaohsiung city, Taiwan

**(* signifies equal contribution compared to the corresponding author)**

**Corresponding author: Cheuk-Kwan Sun, MD, PhD**

Department of Emergency Medicine, E-Da Hospital, Kaohsiung city, Taiwan

No.1, Yida Road, Jiaosu Village, Yanchao District, Kaohsiung City 82445, Taiwan

Telephone: 886-7-6150011 ext. 1007

Fax: +886-7-615-0945

Email: [researchgate000@gmail.com](mailto:researchgate000@gmail.com)

**Supplemental Figure 1.** Search strategies for Medline


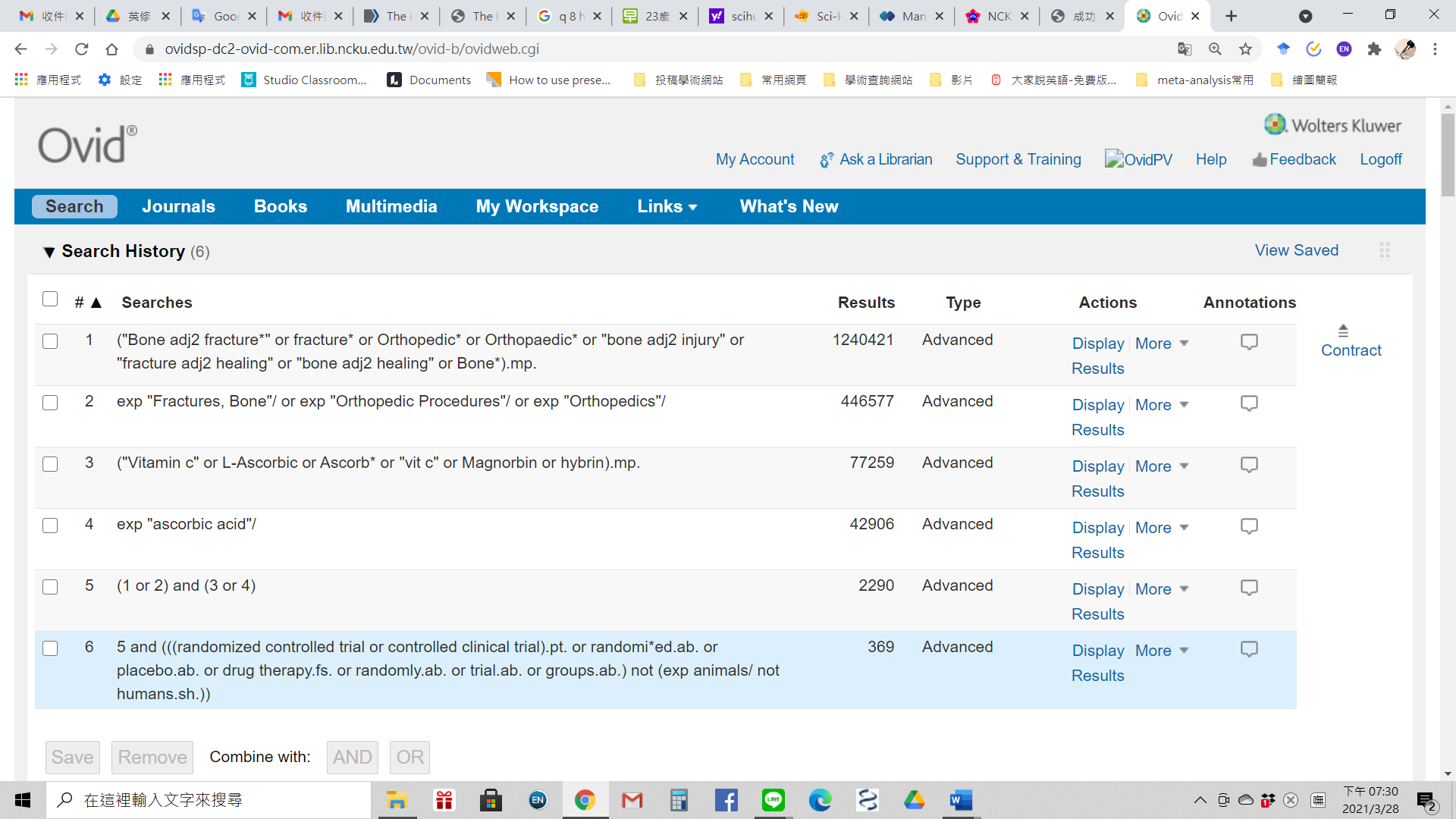


**Supplemental Figure 2. Severity of pain 3–6 months after surgery or trauma**

**
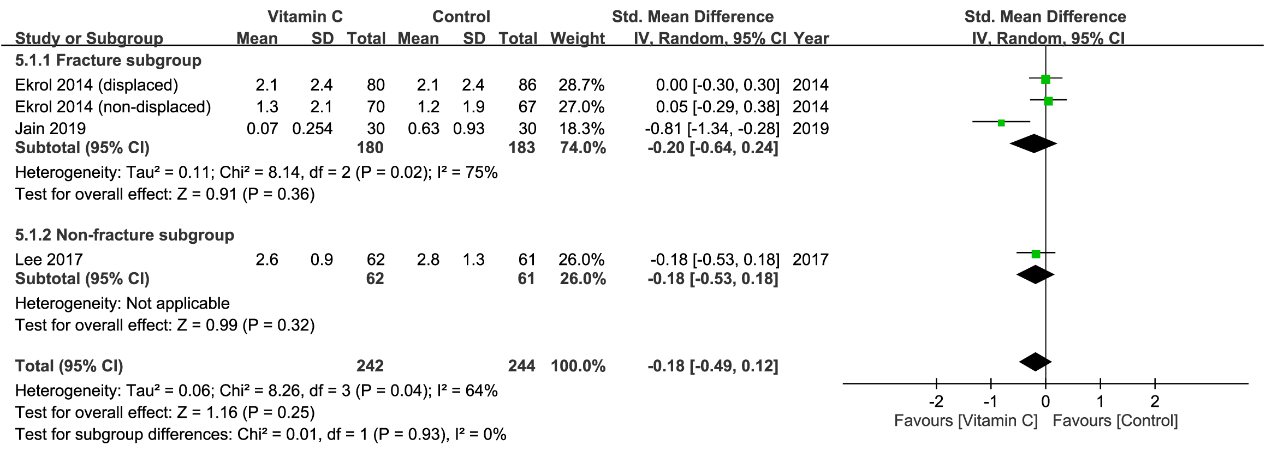
**

**Supplemental Figure 3. Overall complications at 3–6 months after surgery or trauma**

**
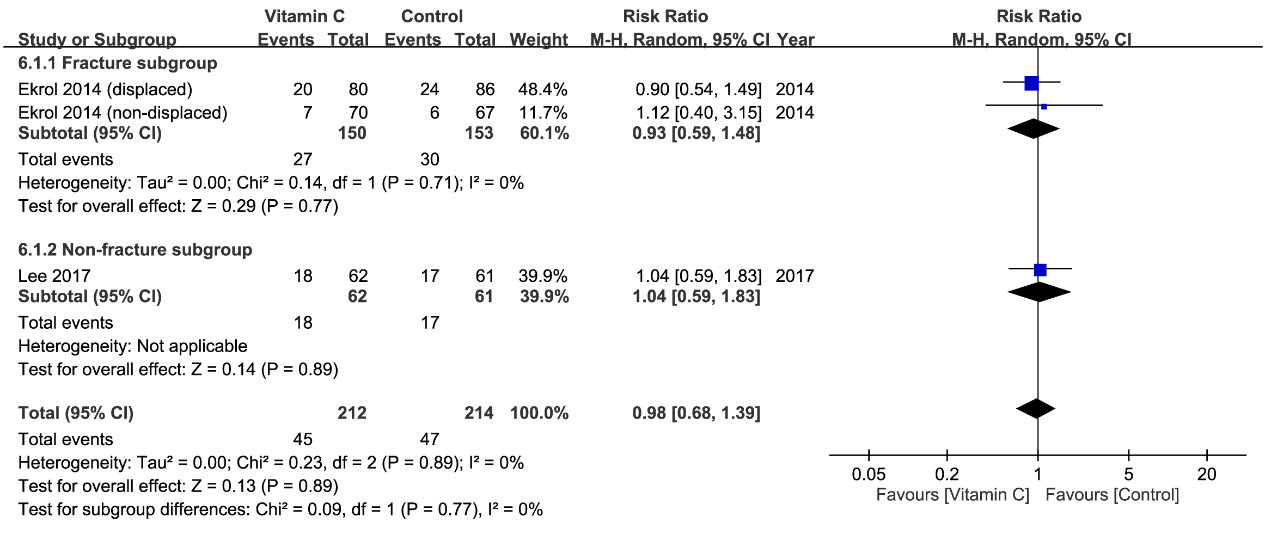
**

**Supplemental Table 1.** Summary of conclusions and limitations of previous meta-analyses on the association between oral Vitamin C supplementation and complex regional pain syndrome I (CRPS I)

| Study name | Conclusion | Limitation of meta-analysis |
| --- | --- | --- |
| Shibuya 2013^1^ | Vitamin C supplementation decreased the risk of CRPS I | Inclusion of two RCTs and two non-randomized studies |
| Evaniew 2015^2^ | Vitamin C supplementation had no impact on the risk of CRPS I | Patients receiving low dose of Vitamin C (200 mg) were not exclude for analysis |
| Meena 2015^3^ | Vitamin C supplementation decreased the risk of CRPS I | Inclusion of two RCTs and one non-randomized study |
| Chen 2016^4^ | Vitamin C supplementation decreased the risk of CRPS | Inclusion of one RCT and two non-randomized studies |
| Aim 2017^5^ | Vitamin C supplementation decreased the risk of CRPS | Inclusion of RCTs, but fixed effect model was used to measure the effects |

CRPS I: complex regional pain syndrome I; RCT: randomized controlled trial

**References**

1. Naohiro Shibuya, Jon M Humphers, Monica R Agarwal, Daniel C Jupiter. Efficacy and safety of high-dose vitamin C on complex regional pain syndrome in extremity trauma and surgery--systematic review and meta-analysis. J Foot Ankle Surg. 2013;52(1):62-6.
2. Nathan Evaniew, Colm McCarthy, Ydo V Kleinlugtenbelt, Michelle Ghert, Mohit Bhandari. Vitamin C to Prevent Complex Regional Pain Syndrome in Patients With Distal Radius Fractures: A Meta-Analysis of Randomized Controlled Trials. J Orthop Trauma. 2015;29(8):e235-41.
3. Sanjay Meena, Pankaj Sharma, Shreesh Kumar Gangary, Buddhadev Chowdhury. Role of vitamin C in prevention of complex regional pain syndrome after distal radius fractures: a meta-analysis. Eur J Orthop Surg Traumatol. 2015;25(4):637-41.
4. Suzan Chen, Darren M Roffey, Charles-Antoine Dion, Abdullah Arab, Eugene K Wai. Effect of Perioperative Vitamin C Supplementation on Postoperative Pain and the Incidence of Chronic Regional Pain Syndrome: A Systematic Review and Meta-Analysis. Clin J Pain. 2016;32(2):179-85.
5. F Aïm, S Klouche, A Frison, T Bauer, P Hardy. Efficacy of vitamin C in preventing complex regional pain syndrome after wrist fracture: A systematic review and meta-analysis. Orthop Traumatol Surg Res. 2017;103(3):465-470.
